# Fatal pulmonary thromboembolism in asymptomatic COVID-19

**DOI:** 10.1007/s11845-021-02735-8

**Published:** 2021-09-05

**Authors:** Gerard Keane, Tony Dorman

**Affiliations:** 1 School of Medicine, Royal College of Surgeons in Ireland, Dublin, Ireland; 2 Department of Histopathology, Beaumont Hospital, Dublin, Ireland

**Keywords:** Asymptomatic, COVID-19, Pulmonary thromboembolism, SARS-CoV-2, Thrombosis

## Abstract

**Background:**

Coronavirus disease 2019 (COVID-19) has claimed the lives of millions of people globally.

**Aims:**

This study aims to identify the pathological findings at autopsy of asymptomatic COVID-19 death, to compare the incidence of acute bilateral pulmonary thromboembolism (ABPTE) in asymptomatic COVID-19 deaths versus non-COVID-19 deaths and to explore the possible pathogenesis of thrombosis in COVID-19. We also consider the place of COVID-19 in the death certification of 4 cases who died from ABPTE.

**Methods:**

This study primarily reviewed post-mortem reports of 6 asymptomatic COVID-19 deaths. Post-mortem reports for the years 2019 and 2020 were also reviewed to establish the incidence of ABPTE. Each post-mortem report was reviewed for gross examination, histology and toxicology findings. A literature review on COVID-19 autopsy findings, COVID-19 pathogenesis, thrombosis in COVID-19 and asymptomatic SARS-CoV-2 infection was also conducted using PubMed.

**Results:**

Of the 6 asymptomatic COVID-19 deaths, 4 died as a result of ABPTE, 1 died of ischaemic and hypertensive cardiac disease caused by coronary artery disease and ventricular hypertrophy and the remaining case died of heart failure due to dilated cardiomyopathy caused by subendocardial fibrosis. There were 2 cases of bilateral pulmonary thromboembolism (BPTE) in 2019 out of 140 post-mortems. Excluding the 4 cases of ABPTE described already, there was 1 case of ABPTE in 2020 out of 156 post-mortems. A literature review on the pathogenesis of thrombosis in COVID-19 highlighted the significant role that the endothelium plays.

**Conclusions:**

Massive pulmonary thromboembolism may be a significant cause of death in asymptomatic COVID-19 infection.

## Introduction

The global spread of the severe acute respiratory syndrome coronavirus 2 (SARS-CoV-2) causing coronavirus disease 2019 (COVID-19) has led to more than 3 million deaths to date [1]. Much research has been published on COVID-19; however, the pathophysiology of the disease is not yet fully understood. We are reminded of the importance of autopsies when new diseases like COVID-19 emerge for which the pathology is unknown [2]. Autopsy is considered the gold standard for identifying the cause of death [3]. However, early in the pandemic, very few autopsies of COVID-19 deaths were performed [4]. In Ireland, autopsies were and are still deemed unsafe to perform due to concerns for the safety of mortuary staff [5]. Under Irish law, unexplained deaths are reportable to the coroner, who can order a post-mortem to be performed [6]. We report 6 unexplained sudden deaths from April and May 2020, all of whom tested positive for SARS-CoV-2 infection, but did not show symptoms of COVID-19 (based on collateral histories from relatives). None were hospitalized, and all were living in the community at the time of death.

Typical symptoms associated with COVID-19 include fever, cough, shortness of breath and loss of taste or smell [7]. However, there is uncertainty around the proportion of asymptomatic infection. One estimate suggests that 33% of people with SARS-CoV-2 infection never develop symptoms. Many asymptomatic individuals have been admitted to hospital with unrelated ailments, which upon testing and imaging show signs of COVID-19 disease including ground glass opacities in the lungs and evidence of pneumonia [8]. These findings suggest that asymptomatic individuals are not immune to the effects of COVID-19, highlighting the importance of conducting autopsies of asymptomatic COVID-19 deaths.

In contrast to Ireland, certain countries including Austria and Germany mandated autopsies of all COVID-19 deaths. Autopsy findings revealed thrombosis to be a major cause of death, but the pathogenesis of this thrombosis was not clear [2, 3]. The cause of death in 4 of our 6 post-mortems was acute bilateral pulmonary thromboembolism (ABPTE). Pulmonary thromboembolism is a recognized complication of severe symptomatic COVID-19 disease [8]. With this in mind, our study aims to identify the pathological findings at autopsy of asymptomatic COVID-19 death, to compare the incidence of ABPTE in asymptomatic COVID-19 deaths versus non-COVID-19 deaths and to explore the possible pathogenesis of thrombosis in COVID-19. We also consider the place of COVID-19 in the death certification of 4 cases who died from ABPTE.

## Materials and methods

### Materials

This study primarily reviewed post-mortem examinations (reports) performed by one pathologist at Dublin City Mortuary of 6 asymptomatic COVID-19 deaths. Autopsies were conducted between 7 April 2020 and 27 May 2020. These included the first 5 COVID-19 autopsies conducted in Ireland [9]. Post-mortem reports from the same facility and pathologist for the years 2019 and 2020 were also reviewed to establish the incidence of ABPTE.

### Methods

Due to the threat of COVID-19 to mortuary staff, limited autopsy, histology and toxicology were performed for these cases. Normal autopsy components not performed included examination of the head (including the brain) and the removal of abdominal and pelvic contents. Contents instead were examined in situ with histology samples taken of the liver, spleen, kidneys and any abnormality identified. Histology samples were also taken from all lung lobes as well as from the left and right heart ventricles. Toxicology specimens were taken from all cases.

Each post-mortem report was reviewed for gross examination, histology and toxicology findings. A review of the current literature on COVID-19 autopsy findings, COVID-19 pathogenesis, thrombosis in COVID-19 and asymptomatic SARS-CoV-2 infection was also conducted using PubMed.

The first 5 cases tested positive for COVID-19 in the community as they were close contacts of symptomatic patients. The sixth case tested positive in the mortuary (testing of all bodies was introduced to Dublin City Mortuary in mid-April 2020). Immunohistochemistry used to confirm COVID-19 infection was not performed as it was not available at the time.

## Results

Patient characteristics and autopsy findings are summarized in Table 1. The median age of the six cases was 64.5 years (range, 36–76), and one-third were women. Toxicology results were non-contributory in all cases. The risk factors of severe illness in COVID-19 include increasing age, cardiovascular disease, hypertension, diabetes mellitus, chronic lung disease, cancer (especially haematologic malignancies, lung cancer and metastatic cancer), chronic kidney disease, obesity and smoking [8]. Case 1 had a kidney infection and hypertension and was being treated for throat cancer. Case 2 had a breast cancer surgery scar, was mildly obese and was a nursing home resident. Case 3 was severely obese. Case 4 had a myocardial infarct 2 years previous and was diabetic, on dialysis and mildly obese. Case 5 was 74 and was receiving daily care at home. Case 6 was 76 and had chest pain for 2 weeks.

**Table 1:** Patient characteristics and autopsy findings

Case number	Age	Sex	Pre-existing medical conditions	Cause of death	Main pathological findings
1	62	Male	Throat cancer, kidney infection, hypertension	ABPTE	ABPTE. Acute diffuse bilateral pulmonary oedema. Congested liver. Gallbladder mucocoele. Severe benign prostatic hyperplasia. Suspect pelvic deep venous thrombosis. Negative for residual throat carcinoma
2	67	Female	Breast cancer (scar), mild obesity, schizophrenia, hypothyroid, nursing home resident	ABPTE	ABPTE. Left leg 2 cm thicker. Left calf erythema. Negative for residual breast carcinoma
3	36	Female	Severe obesity	ABPTE	ABPTE. Left calf swelling. Dilated left and right ventricles. Acute pulmonary oedema
4	43	Male	2-year-old myocardial infarct, diabetic on dialysis, mild obesity	Ischaemic and hypertensive heart disease	Old healed myocardial infarct (left anterior descending). Moderate cardiomegaly (545 g). Severe left ventricular hypertrophy. Severe coronary artery disease
5	74	Male	Care recipient	ABPTE	ABPTE. Mild cardiomegaly (465 g). Left and right ventricular hypertrophy. Mild degenerative mitral valve. Severe coronary artery disease. Severe aortic atheroma. Suspect pelvic deep venous thrombosis. Pulmonary oedema. Mild distal oesophagitis. Congested liver. Mild benign prostatic hyperplasia. Small spleen (60 g)
6	76	Male	2 weeks of chest pain, left hospital recently	Congestive cardiac failure due to dilated cardiomyopathy due to subendocardial fibrosis	Left ventricle diffuse subendocardial fibrosis with ventricle dilatation. Mitral and aortic valve enlargement. Severe cardiomegaly (665 g). Pulmonary oedema. Features of congested cardiac failure

### Autopsy

**Fig. 1:**
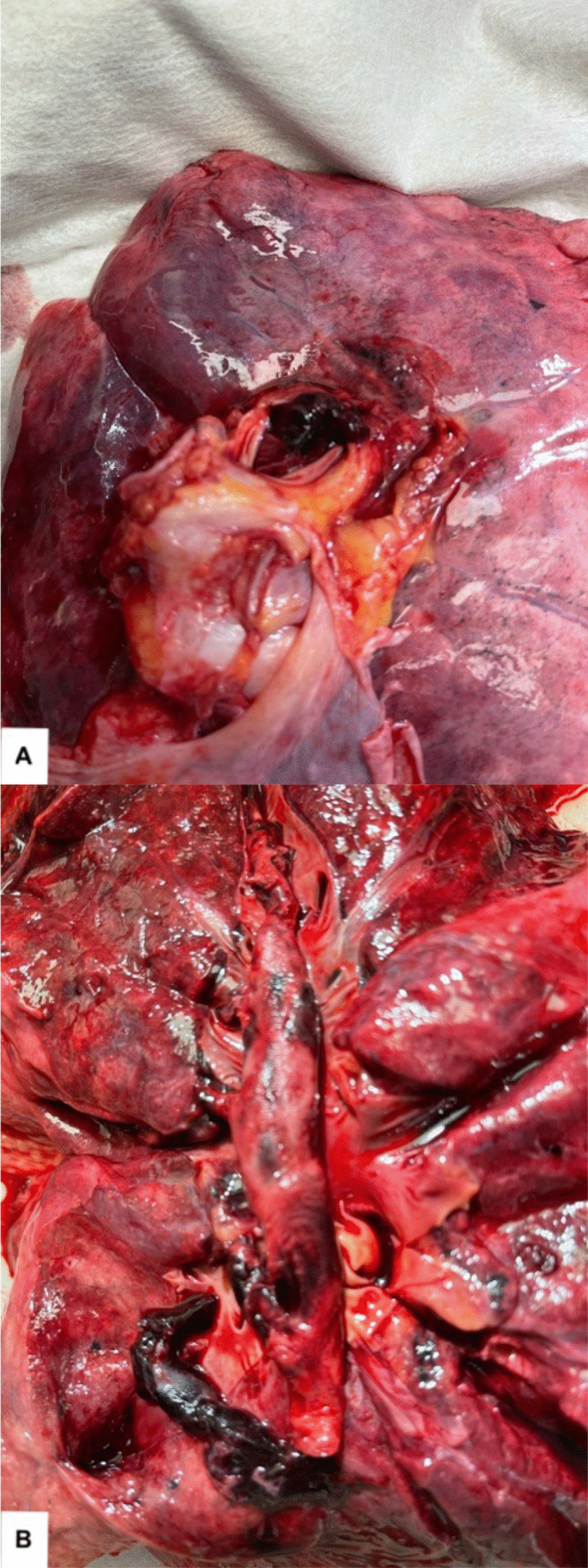
Acute bilateral pulmonary thromboembolism (ABPTE). **A** Pulmonary artery occluded by thromboembolism at lung hilum. **B** Open cross-section of the pulmonary artery tree showing occlusion by thromboembolism

The cause of death in 4 of the 6 cases (1, 2, 3 and 5) was ABPTE (Fig. 1). Case 4 died of ischaemic and hypertensive heart disease and case 6 died of congestive cardiac failure. Of those who died of ABPTE, 2 cases (1 and 5) had suspect pelvic deep venous thrombosis with either mild or severe benign prostatic hyperplasia. The other cases (2 and 3) had left calf swelling.

Significant cardiac findings were seen in cases 3, 4, 5 and 6. These included cardiomegaly (cases 4, 5 and 6), coronary artery disease (cases 4, 5 and 6), ventricular dilation (cases 3 and 6), ventricular hypertrophy (cases 4 and 5), valve degeneration (cases 5 and 6) and aortic atheroma (cases 5 and 6). Case 6 had subendocardial fibrosis, fibrosis and inflammation of the pericardium (pericarditis) as well as conjunctival swelling.

As expected, pulmonary oedema (cases 1, 3, 5 and 6) and congested liver (cases 1, 5, and 6) were also seen.

### Histology

Targeted histology was conducted for cases 1, 2, 3 and 4. Findings are summarized in Table 2.

**Table 2:** Histological findings in cases 1–4 (not performed in vases 5 and 6)

Case number	Lung	Heart	Liver	Kidney	Spleen
1	Occasional micro-thromboemboli, mild focal emphysema, pulmonary oedema foci, mild emphysema	Mild hypertensive nuclear changes, mild fibrosis	Mild fatty change, acute venous congestion	Mild/moderate arteriosclerosis, mild fibrosis	Congestion of red pulp
2	Moderate emphysema (fibrosis), pulmonary oedema	Hypertensive nuclear changes, severe focal fibrosis, focal plasma cell pericarditis			
3	Acute pulmonary oedema, foci of anthracosis with adjacent mild chronic inflammation, some nuclear enlargement without inflammation, focal pulmonary acute thromboembolism associated with early acute bronchopneumonia, type 2 pneumocyte hyperplasia, occasional hyaline membranes	Myocardium: mild hypertensive nuclear changes Pericardium: very occasional lymphocytes			
4	Mild emphysema, heart failure cells, mild chronic inflammation				

In the lungs, emphysema was seen in cases 1, 2 and 4. Mild chronic inflammation was seen in cases 3 and 4. At least one micro-thromboembolism was seen in cases 1 and 3. Case 3 showed anthracosis, type 2 pneumocyte hyperplasia and early acute bronchopneumonia. In addition, occasional hyaline membranes were also identified.

In the heart, hypertensive nuclear changes were seen in cases 1, 2 and 3. Fibrosis was seen in cases 1 and 2. Lymphocytes were seen in the pericardium of cases 2 and 3. Case 2 had focal pericarditis.

Case 1 had additional histology performed which included mild fatty change of the liver as well as mild fibrosis and arteriolosclerosis of the kidney.

### Retrospective post-mortem review

A retrospective review of all 140 post-mortems carried out by the same pathologist at Dublin City Mortuary in the year 2019 showed two cases of BPTE, a less severe form of ABPTE. The year 2020 showed that of 156 post-mortems, there were 5 cases of ABPTE (the four cases in this study and a fifth case in a COVID-19-negative patient in December 2020).

### Pathogenesis of thrombosis in COVID-19

A literature review on the pathogenesis of thrombosis in COVID-19 highlighted the significant role of the endothelium in eliciting the hypercoagulable state seen in COVID-19 [10–15].

**Fig. 2:**
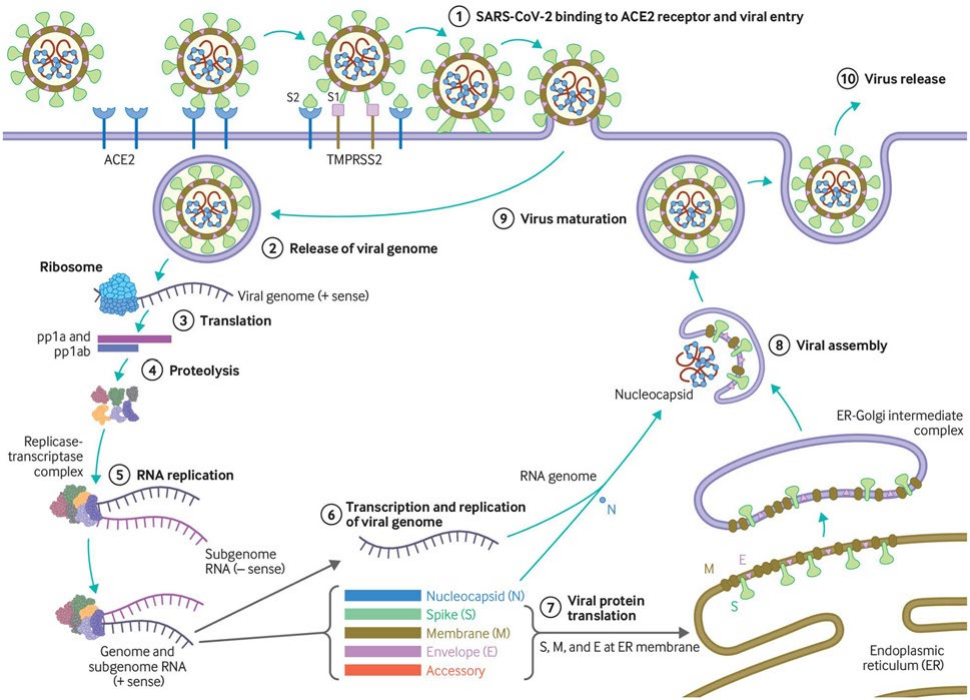
SARS-CoV-2 entry, replication and exit. Reproduced from Virology, transmission, and pathogenesis of SARS-CoV-2, Cevik M, Kuppalli K, Kindrachuk J, et al., 371, 3862, 2021 with permission from BMJ Publishing Group Ltd

Figure 2 demonstrates how SARS-CoV-2 enters a host cell via the transmembrane protein, angiotensin-converting enzyme 2 (ACE2), replicates and exits in order to infect other host cells, killing the host cell in the process [10, 11]. Crucially, ACE2, the means by which SARS-CoV-2 infects cells, is expressed by (among others) type II pneumocytes and endothelial cells that line arteries and veins. During homeostasis, ACE2 functions to lower blood pressure by converting angiotensin II (a vasoconstrictor) to angiotensin 1–7 (a vasodilator). However, ACE2 is internalized during viral entry and in turn downregulated, increasing the amount of circulating (vasoconstricting) angiotensin II.

**Fig. 3:**
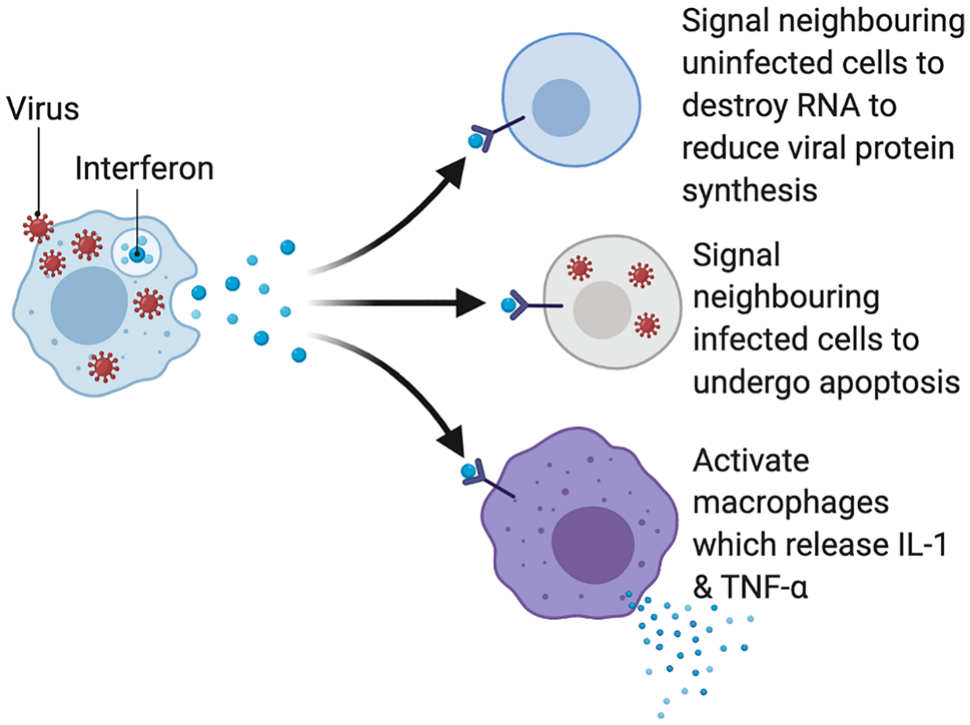
Host cell inducing interferon production in order to interfere with viral replication

Figure 3 shows how viral entry induces the host cell to produce interferons as part of the innate immune response to try to shut down viral replication in that cell and neighbouring cells. Interferons recruit macrophages to attack the virus which release proinflammatory cytokines IL-1 and TNF-α. Neighbouring cells are instructed to undergo apoptosis or to destroy RNA, all to reduce viral spread. IL-1 and TNF-α are also released during host cell death and apoptosis. If, however, the virus is successful in replicating virus particles (virions), these virions exit the cell and can enter the bloodstream via alveolar capillaries. Now the virus can infect endothelial cells.

**Fig. 4:**
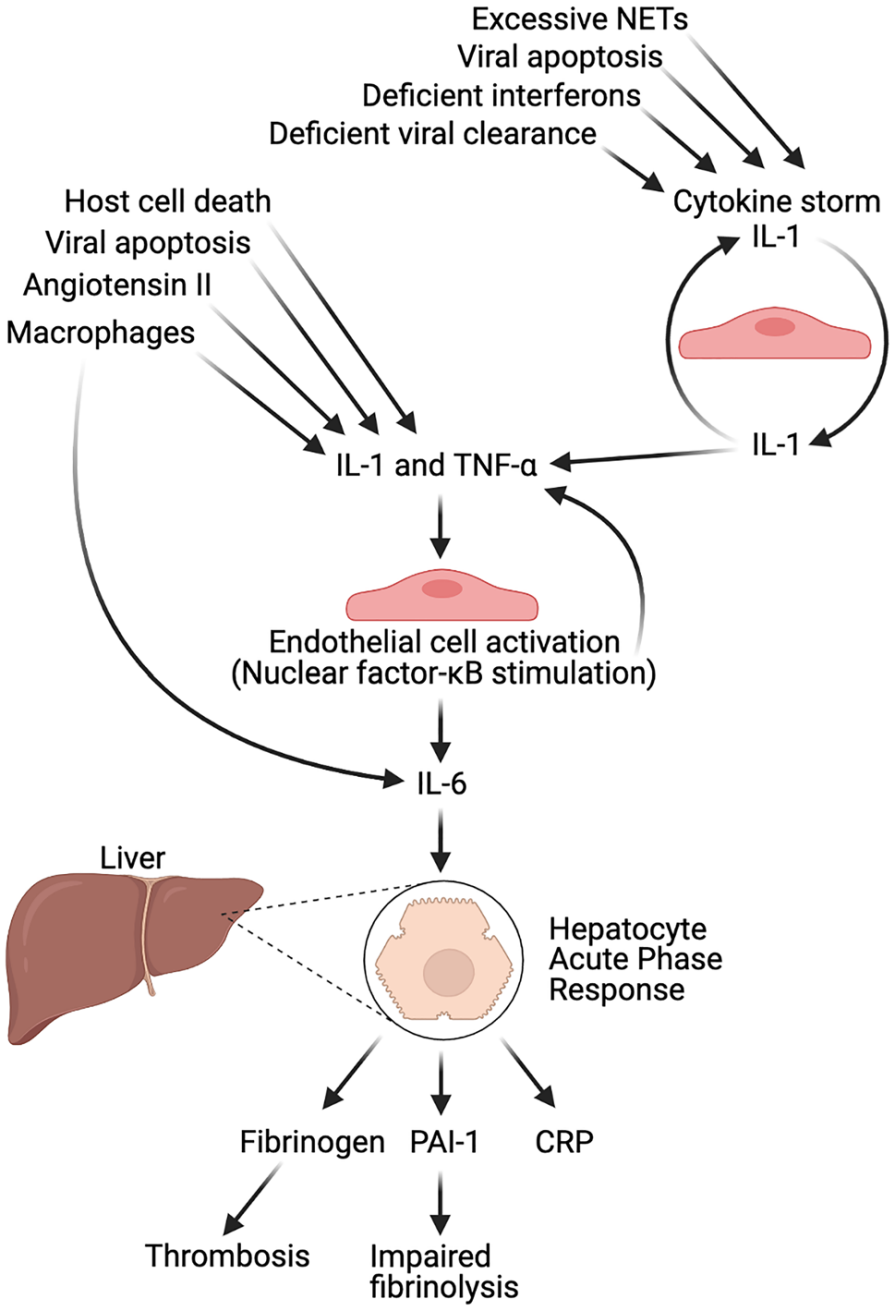
IL-1 and TNF-α production promoting thrombosis

Figure 4 shows the effect that IL-1 and TNF-α production has on thrombosis pathogenesis in COVID-19. IL-1 and TNF-α target uninfected endothelial cells as they contain the proinflammatory transcriptional hub, nuclear factor-κB, which results in more of these proinflammatory cytokines being produced. Angiotensin II described previously also stimulates nuclear factor-κB. IL-1 causes endothelial cells to produce IL-6, which acts on the liver to induce the acute phase response. IL-6 is also produced by macrophages. The liver produces fibrinogen, plasminogen activator inhibitor-1 (PAI-1) and C-reactive protein (CRP) as a result. Fibrinogen is a precursor of fibrin used to form thrombi. PAI-1 inhibits the activation of plasminogen into plasmin, which is responsible for fibrinolysis.

The term *cytokine storm* has been used abundantly when describing severe COVID-19 infection. In the context of the endothelium, a cytokine storm is when IL-1 produced by endothelial cells induces its own gene expression, causing it to continuously produce, creating a procoagulant state in the bloodstream (Fig. 4). IL-1 also induces the production of TNF-α, and TNF-α in turn induces the production of IL-1. A number of factors can pre-empt a cytokine storm in COVID-19 including impaired viral clearance, a low level of type 1 interferons, excessive neutrophil extracellular traps (NETs) (which are usually anti-viral) and viral apoptosis with the associated release of proinflammatory cytokines (pyroptosis).

**Fig. 5:**
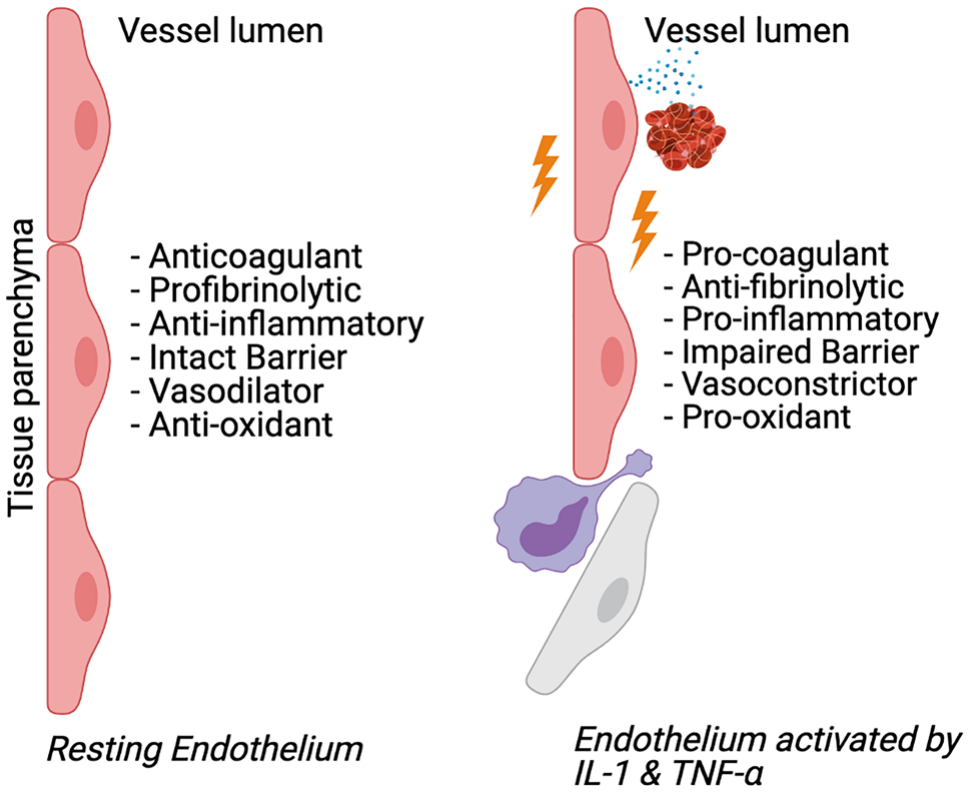
The endothelium during homeostasis (left) and activated by IL-1 and TNF-α (right)

The left part of Fig. 5 lists the properties of a normal endothelium during homeostasis. The right part of Fig. 5 shows what happens to the endothelium when infected and activated by IL-1 and TNF-α. During homeostasis, the endothelium is both anti-coagulant and profibrinolytic in nature. However, viral infection of endothelial cells can activate subendothelial tissue factor, which acts as the spark to start the coagulation cascade. Endothelial cells also store von Willebrand factor, which can be released, encouraging platelet aggregation and eventual clot formation. Under the same proinflammatory stresses, endothelial cells can release PAI-1, which inhibits fibrinolysis. The combination of prothrombic acute phase reactants produced by the liver and the procoagulant effects of IL-1 and TNF-α on endothelial cells causes an imbalance between thrombosis and fibrinolysis, resulting in excessive clot formation.

## Discussion

### Post-mortem examinations

All cases in our autopsy study, on the surface, had at least one risk factor for severe COVID-19 infection. Autopsy and histological examination revealed additional risk factors. Case 1 had mild emphysema as well as arteriosclerosis and fibrosis of the kidneys. Case 2 had hypertensive nuclear changes and fibrosis of the heart as well as pericarditis. Case 3 had chronic inflammation and anthracosis of the lungs as well as dilated cardiomyopathy and hypertensive nuclear changes of the heart. Case 4 had emphysema and chronic inflammation of the lungs as well as ventricular hypertrophy and severe coronary artery disease. Case 5 had ventricular hypertrophy, coronary artery disease and aortic atheroma. Case 6 had significant heart disease. In addition, cases 2 and 3 displayed common non-specific histological findings often seen in COVID-19 and other viral infections [16]. Case 3 displayed type II pneumocyte hyperplasia, nuclear enlargement and occasional hyaline membranes in the lungs. Case 3 also displayed lymphocytic infiltration of the pericardium as did case 2. The combination of known risk factors, autopsy findings and histology findings underlines the fact that all cases in our study were at risk of severe COVID-19 infection despite not displaying symptoms.

The cause of death in 4 of the 6 cases was ABPTE. Several other autopsy studies of symptomatic patients report ABPTE or pulmonary thrombus to be a significant cause of death in COVID-19 patients [2-4, 17, 18]. ICU studies of COVID-19 patients showed the incidence of pulmonary embolism to be twice as frequent compared to a control group [19], while 49% of COVID-19 ICU patients displayed thrombotic complications [20]. In contrast, our retrospective review of 2 years’ worth of post-mortems showed ABPTE to be a rare event in non COVID-19 deaths. These findings suggest that pulmonary embolism is a major contributory factor in COVID-19 deaths.

There have been a handful of papers written about pulmonary thrombus or thromboembolism in asymptomatic COVID-19 infection [21–25]. These papers mostly describe individuals who were initially asymptomatic but later became symptomatic. However, the cases in our report all remained asymptomatic. One paper describes a case of fatal acute bilateral pulmonary thrombus in an individual who remained asymptomatic [24]. To our knowledge, this is the first report of fatal ABPTE in a case of asymptomatic COVID-19 infection.

### Death certification

In Ireland, of those with COVID-19 who died of acute respiratory distress syndrome, COVID-19 was documented as a causative condition in part 1 of the death certificate. After detailed discussions between the coroner and pathologist, in the 4 cases of ABPTE, a decision was made to record COVID-19 as a contributory condition in part 2 of all 4 death certificates. Where COVID-19 lies in the death certification process requires further research. However, it could be argued, based on our initial observations and the data from the literature we cite, that going forward, in the case of asymptomatic COVID-19 deaths attributed to ABPTE, COVID-19 should be documented as a contributory condition. Whether COVID-19 can be documented as a causative condition of ABPTE requires confirmation from larger statistically significant studies. Perhaps when the COVID-19 pandemic has subsided and mandatory autopsy testing is no longer required, cases of ABPTE should continue to be tested for COVID-19.

In summary, our limited study of 6 asymptomatic COVID-19 deaths, related retrospective review of past post-mortems and literature review on the pathogenesis of thrombosis in COVID-19 have shown that thrombosis and massive thromboembolism may be a significant cause of death.

## Conflict of interest

The authors declare no competing interests.
